# Gauging mixed climate extreme value distributions in tropical cyclone regions

**DOI:** 10.1038/s41598-022-08382-y

**Published:** 2022-03-17

**Authors:** J. G. O’Grady, A. G. Stephenson, K. L. McInnes

**Affiliations:** 1 CSIRO Oceans and Atmosphere, Melbourne, Australia; 2 DATA61, Melbourne, Australia

**Keywords:** Statistics, Climate sciences, Natural hazards

## Abstract

In tropical cyclone (TC) regions, tide gauge or numerical hindcast records are usually of insufficient length to have sampled sufficient cyclones to enable robust estimates of the climate of TC-induced extreme water level events. Synthetically-generated TC populations provide a means to define a broader set of plausible TC events to better define the probabilities associated with extreme water level events. The challenge is to unify the estimates of extremes from synthetically-generated TC populations with the observed records, which include mainly non-TC extremes resulting from tides and more frequently occurring atmospheric-depression weather and climate events. We find that extreme water level measurements in multiple tide gauge records in TC regions, some which span more than 100 years, exhibit a behaviour consistent with the combining of two populations, TC and non-TC. We develop an equation to model the combination of two populations of extremes in a single continuous mixed climate (MC) extreme value distribution (EVD). We then run statistical simulations to show that long term records including both historical and synthetic events can be better explained using MC than heavy-tailed generalised EVDs. This has implications for estimating extreme water levels when combining synthetic cyclone extreme sea levels with hindcast water levels to provide actionable information for coastal protection.

## Introduction

Coastal practitioners (e.g. researchers, engineers, builders and managers) require extreme sea level exceedance probabilities to design coastal defence structures and coastal zone management to avoid losses now and into the future^1–7^. Tide gauges record the extreme water levels from passing storms coinciding with astronomical tide and seasonal sea levels (steric and barotropic). In tropical cyclone (TC) regions, tide gauge or numerical hindcast records are usually of insufficient length to have sampled sufficient TC-induced extreme water level events from which robust event statistics can be evaluated for coastal impact studies^7–12^. Tide gauge records can be over 100 years in length (e.g. Honolulu and Galveston in the US), but for many TC regions, such as those situated in the Pacific islands, record are only a few decades in length^13^. Numerical hydrodynamic models forced by atmospheric reanalysis can generate a multi-decade long record of sea level at, and more importantly away from, tide gauge locations to provide greater coastal coverage^14–16^. While TC-driven sea level events are captured in tide gauge and hindcast records, they are under sampled. TC events which could have had different characteristics (track and/or intensity) or could have coincide with different contributors to extreme sea levels, such as the stage of the tide are not captured in the tide gauge record. Furthermore, the observed TC could have been more damaging to coastal communities away from the tide gauge, which has been addressed in synthetic and statistical modelling studies at the regional to global scale^17–19^. Synthetic tracks have been used to evaluate a full range of TC characteristics over timescales of thousands of years, to assess ‘what if’ a TC occurred with different characteristics^3,8,11^. Synthetic simulations also help understand the so-called ‘grey swan’ high impact events which might be expected^20^ and draw attention to ‘black elephant’ events that are expected but ignored^21^.

Extreme value analysis (EVA) (see Methods) enables the probabilistic estimates of extreme sea levels via a parametric Equation^22^. The Gumbel EVD utilises two parameters, the location and scale parameter, to estimate extremes. The GEV EVD includes a third parameter, the shape parameter, to better capture extreme behaviour in empirical data^23,24^. Analysis of tide gauge data at TC locations have found a positive generalised extreme value (GEV) shape parameter, indicating a heavy (or long) tailed distribution^23,25^. Heavy tailed distributions in TC locations have also been observed in surface wind-wave studies^24,26^. Empirical events near the 90% confidence interval of GEV distribution have been suggested as a mixed response from TC and non-TC events^19^. Estimates of extreme water levels from EVA typically differ between multi-decade tide gauge (or hindcast) records and multi-millennial synthetic records^7,8,27^. To estimate extreme water levels from two populations of extremes, Haigh et al.^8^ sampled the maximum of the two EVDs while Dullaart et al.^7^ and Smith et al.^27^ empirically resampled the two populations. The unifying of different storm-driven event populations has been studied for extreme winds using mixed mechanisms (e.g. Cook^28^; Gomes and Vickery^29^). We introduce the parametric MC EVD, formulated from two Gumbel EVDs, which gives a four parameter EVD. Including more than four parameters in a EVD may find a closer model fit to the empirical data, but there needs to be a physical explanation of drivers of the separate populations (Gomes and Vickery^29^). Parametric models provide the ability to easily compute confidence intervals of probabilistic estimates. Long records can be generated using synthetic TCs, and these can be analysed by non-parametric methods, however a parametric model can more naturally combine both aspects of the mixed climate and can therefore produce inferences that sensibly combine tide gauge information with synthetic TC data.

In this study, we show that mixed climates are measurable in tide gauge records in locations affected by tropical cyclones with sufficiently long observational records and we develop a parametric mixed-climate Extreme Value Distribution to describe such scenarios. In the next section, we show that extreme water level measurements in multiple tide gauge records, some with length greater than 100 years, exhibit behaviour consistent with two combined populations, TC and non-TC. On a return period plot this exhibits the form of a piecewise smooth function with two distinct pieces, which we hereafter refer to as an articulated form. We provide a novel formulation to account for two EVDs in a single continuous mixed climate (MC) EVD equation (see Methods). The MC EVD equation is applied to the observational records and statistical simulations are performed to compare the stability in detecting MC and heavy-tailed generalised EVDs. The use of the MC EVD is also demonstrated by combining measured extreme sea levels in relatively short records with hydrodynamically-modelled extreme water levels from populations of synthetically developed TCs.

## Results

### Identifying mixed climates in tide gauge observations

**Figure 1:**
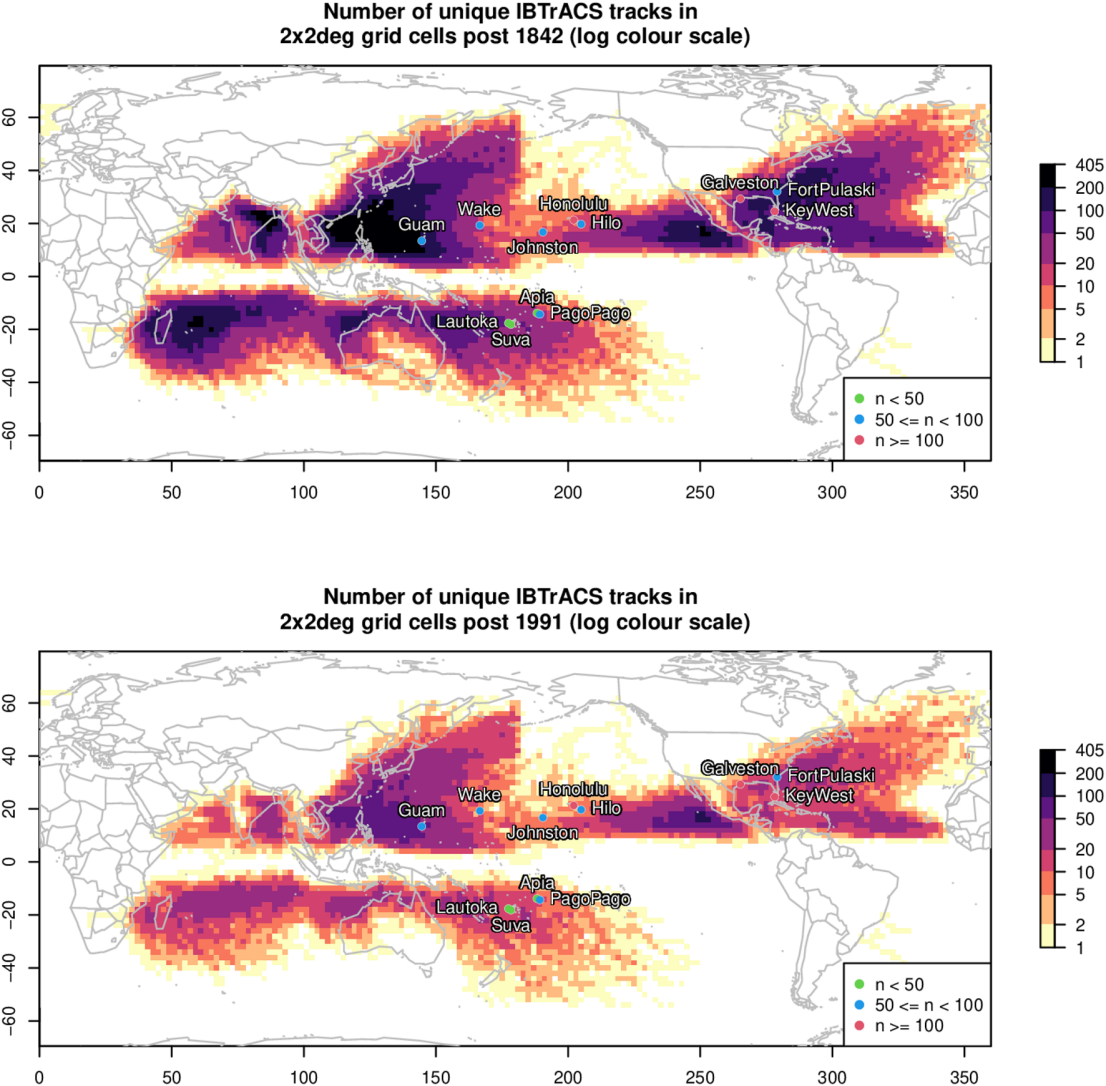
Maps showing location record length (n years) of tide gauges used in this study overlayed on the gridded storm track density (coloured by number of storms, log colour scale) in the IBTrACs v04r00 dataset. Top: track count since 1842, bottom: track count since 1991.

**Figure 2:**
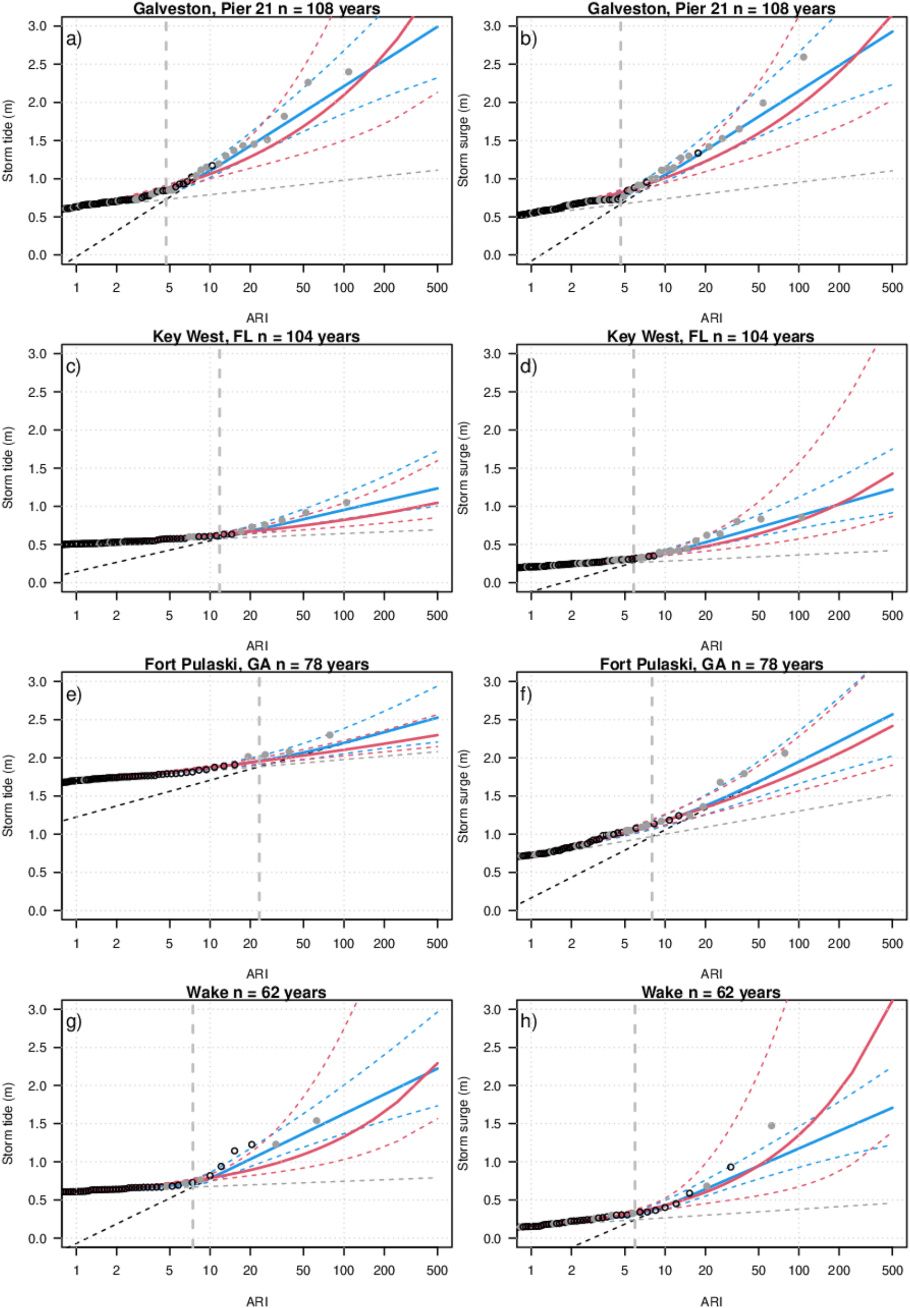
Tide gauge extreme storm tide (left column) and storm surge (right column). Empirically ranked annual maximum for non-TC events (black circles) and TC events (grey points) with fitted MC Gumbel (grey and black dashed lines), continuous MC (blue lines) and GEV (red lines) EVDs with 90% confidence intervals (dashed curves). Vertical thick grey dashed line indicates the intersection of the two MC Gumbel EVD.

The TC impacted tide gauge sites which we consider to be the longest records in the database are mapped and colour coded for record length and overlaid on gridded storm-track occurrence in Fig. 1. The storm tide return levels at two tide gauges spanning more than 100 years in the Gulf of Mexico (Galveston and Key West), along with one spanning more than 50 years in the West-Atlantic Ocean (Fort Pulaski) and one in the Western Pacific Ocean (Wake) are presented in Fig. 2a,c,e,g (other locations are presented in the supplementary report Fig. S1). Major TC events at these locations have been studied in detail previously^30,31^. In Fig. 2, the generalised EVD closely fits the empirically ranked storm tide data for Galveston (Fig. 2a), however, it consistently underestimates return levels at Key west (Fig. 2c), Fort Pulaski (Fig. 2e) and other locations (supplementary report Fig. S1). Rather than a curved generalised EVD line, the empirically ranked annual maxima displays a noticeable articulated form, i.e. two lines meeting together, represented by the continuous MC EVD (Fig. 2). The additional parameter in the MC model allows a closer fit to the empirical data and therefore a tighter model confidence limits at all sites when compared to the GEV confidence limits.

The Akaike information criterion (AIC)^32^, was used to compare the goodness of fit for the three parameter GEV and four parameter MC EVD (Table 1). Lower values of AIC indicate better models, in the sense that the model fit is better relative to the number of model parameters. The AIC table indicates that the MC EVD is a better model at tide gauge sites where there is a more noticeable articulation of two populations of extremes in the empirical data. At some sites the two populations could not be distinguished, in particular the location parameters of both Gumbel distributions were occasionally estimated to be equal. This may be due to the nature of the site, or that the amount of data was not sufficient to make the two populations identifiable. For such data, where there is less noticeable articulation, the AIC table indicates that the GEV is a better model, which is to be expected. In summary, the MC EVD is an important modelling tool at sites where the data enables the articulation to be identified.

**Table 1:** AIC estimates for the tide gauge GEV and MC EVD fits. Bold numbers indicate lower (more negative) AIC values (i.e. better models).

Site	Number of years	Storm tide		Storm surge	
		GEV AIC	MC AIC	GEV AIC	MC AIC
Honolulu	112	**−406.18**	−400.92	**−335.95**	−332.17
Galveston	108	−64.44	−**67.5**	−44.14	−**46.68**
Key West	104	−319.39	−**322.61**	−251.11	−**252.13**
Fort Pulaski	78	−**150**	−149.5	−**17.31**	−16.23
Hilo	76	−**249.98**	−245.08	−226.07	−**229.55**
Pago Pago	66	−**303.48**	−301.28	−194.83	−**200.28**
Wake	62	−158.89	−**165.94**	−**109.17**	−103.48
Guam	61	−**227.86**	−225.1	−**194.46**	−192.83
Johnston	61	−**106.91**	−104.78	−**77.1**	−75.99
Suva	43	−**147.71**	−142.59	−109.86	−**112.16**
Apia	40	−**139.81**	−137.93	−**88.58**	−86.55
Lautoka	26	−91.09	−**91.39**	−59.69	−**60.47**

The articulated form is equally noticeable in storm surge return levels (with tide removed) at these locations in Fig. 2b,d,f,h, highlighting the influence of different meteorological drivers on the extremes. Figure 1 (and supplementary Tables S5 and S6) show the highest annual maxima are identified as TCs (Hurricanes), and a few lower annual recurrence interval (ARI) events as tropical depressions. We note here that storm identification (supplementary Table S4) can sometimes be mismatched, as classification is made subjectively by expert meteorologists, and storms pre-1980 have limited observations. Not all locations display the articulated form for storm tide (Fig. S1), e.g. Lautoka, Suva, Apia, Pago Pago, Honolulu, which is likely due to their short length and low numbers of proximate TCs. However, the articulated form for storm surge (with tide removed) is shown at all locations besides Guam and Apia, while Johnston and Suva have sub annual intersections (Fig. S2). Here Guam experiences some of the most frequent occurring TCs on the globe (Fig. 1) so all annual maxima are dominated by TCs, while Apia sits on the fringe of the mapped TC occurrences (Fig. 1), and has a relatively short record, so at these locations, two distinct populations could not be detected from the recorded annual maxima.

**Figure 3:**
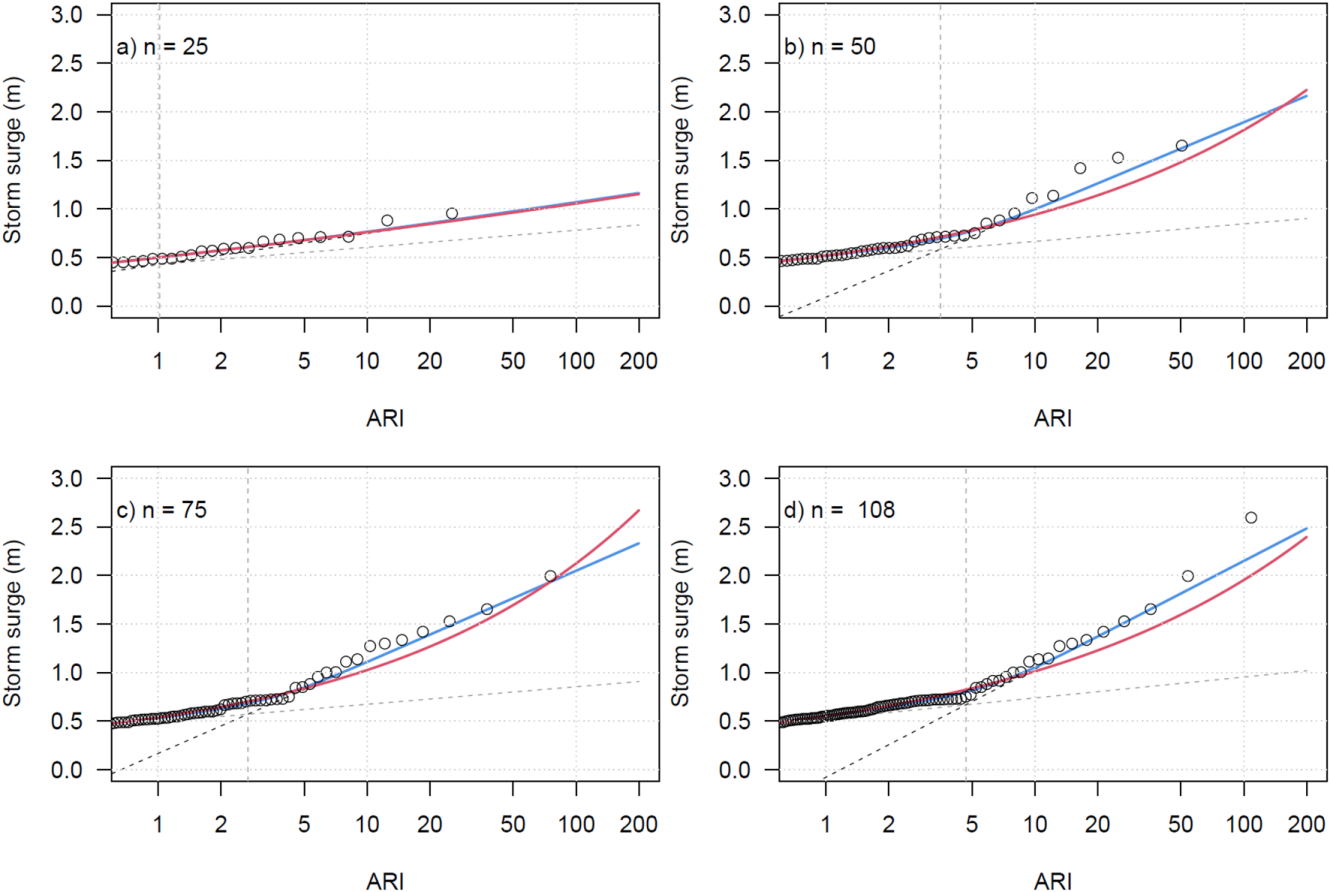
Galveston tide gauge storm surge (tide removed) EVD sensitivity to record length ($\mathrm{n}$). Empirically ranked annual maximum storm tide (black circles) with fitted MC Gumbel (grey and black dashed lines), continuous MC (blue line) and GEV (red line) EVDs using the first n years of annual maxima, a) n = 25, b) n = 50, c) n = 75 and d) n = 108. Vertical thick grey dashed line indicates the intersection of the two MC Gumbel EVDs.

To investigate the importance of record length ($n$) in exhibiting the MC behaviour, the extreme value fits for two of the top three longest records, Galveston and Key West, are plotted for different record lengths in Fig. 3 and Fig. S3. At both locations, the MC EVD is not noticeable when the record length is the first $n=$ 25 years, and the shape parameter of the GEV EVD is near zero. However, for records representing longer time spans, the jointed form emerges and is better represented by the MC EVD than the GEV EVD. Starting with the last year and extending it back further in time does change the shape of the curves, however as expected the mixed climate form is more evident for longer records.

### Combining tide gauge observations with synthetic records

**Figure 4:**
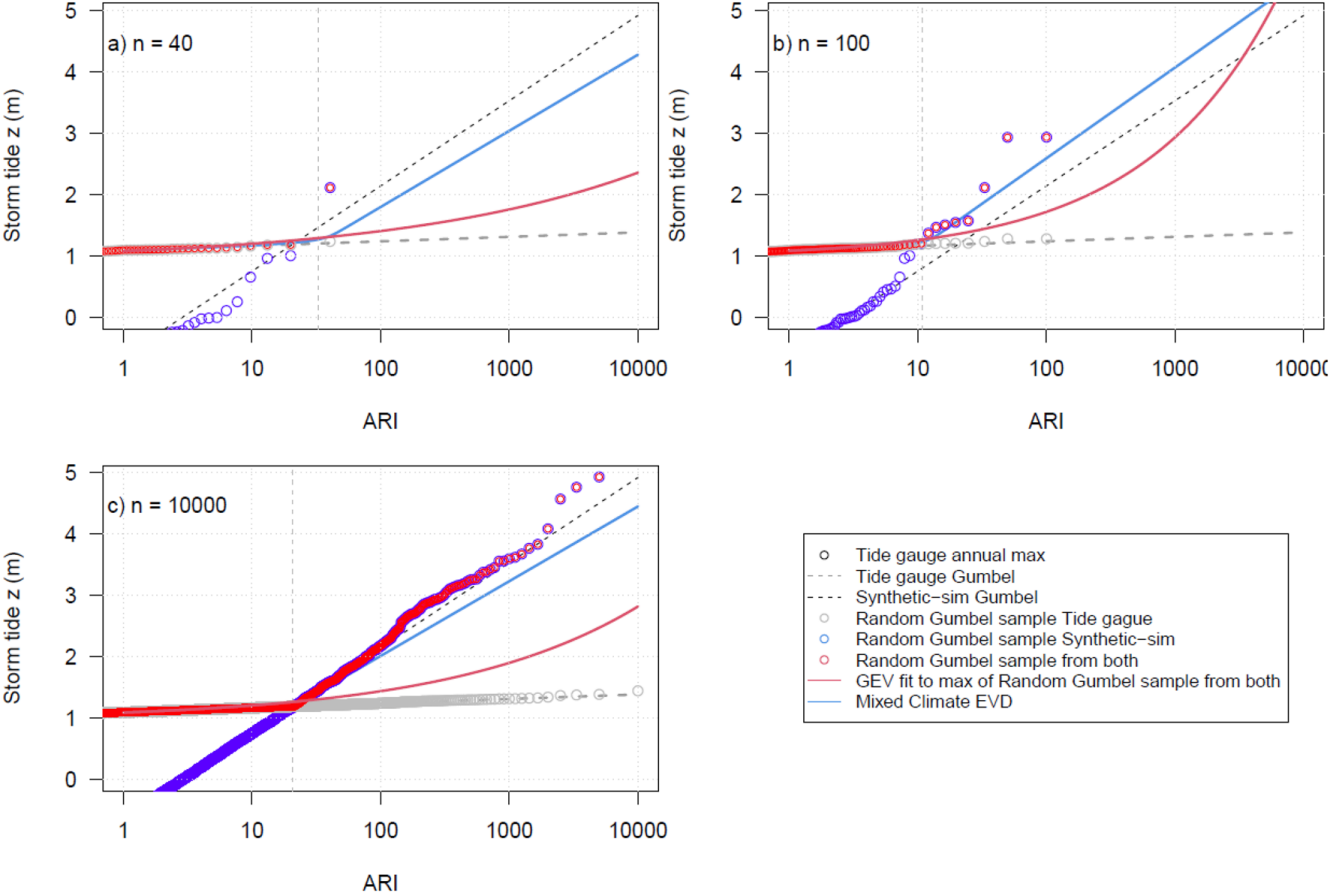
Random sampling of the mixed-climate EVDs for record length (n) equals 40 years (top left), 100 years (top right) and 10,000 years (bottom left) for Lautoka, Fiji.

**Figure 5:**
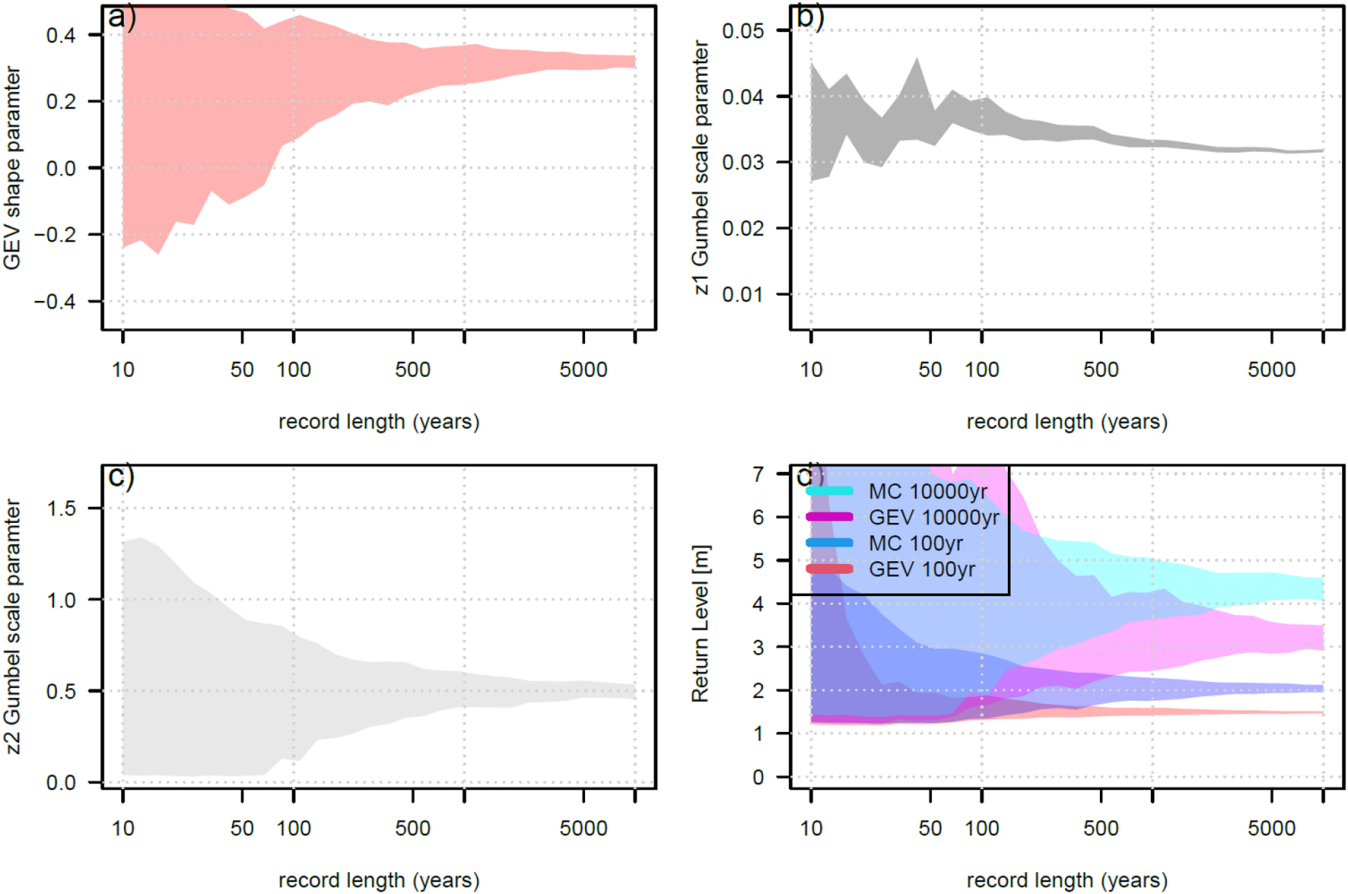
Stability plot for the generalised (GEV) and mixed climate (MC) EVD parameters and return levels for increasing record length (random samples) for storm tide at Lautoka, Fiji. X-axis on a log scale. 90% confidence bounds calculated from 100 Monte Carlo simulations for each record length.

To investigate why some locations do not show the MC articulated form, the measured and synthetic records^11,33^ at Lautoka are randomly sampled to generate a long-term population (Suva and Apia presented in the supplementary material Fig. S4-S7 and Tables S1). Lautoka is presented as it has a short tide gauge record (26 years) and a relatively large tidal range compared to the TC-identified storm surge events including named TC event Evan 2012, Gavin 1997, Kina 1992 and Mick 2009 (Fig. S2), meaning the same TCs are less likely to influence the annual maximum storm tide extremes (Fig. S1). Fig. 4 shows the randomly sampled annual maximum from the tide gauge and synthetic EVDs for $n=$ 40, 100 and 10,000 years. Compared to the mixed climate, the GEV distribution underestimates the model-derived synthetic cyclone data at higher ARIs. Fig. 5d presents how the 10,000 and 100 year ARI return levels stabilise after the record length is 100 years, and that the GEV EVD typically underestimates the assumed mixed climate model derived data. This stability is reflected in the GEV shape parameter (Fig. 5a), where after n = 100 years only positive parameter estimates are produced, and the range of the Gumbel scale parameters of both MC population (Fig. 5b,c) narrows considerably.

## Discussion

The mixed climate extreme value distribution (MC EVD) appears to better represent the empirical record of extreme water levels where TC events are abundant, such as in Galveston and Key West. In locations where fewer TCs are recorded, the MC EVD can be used to connect the probability of two records of extreme water levels, one relying on what has occurred (tide gauge records) and one on what could be possible (synthetic TC simulations). We demonstrate that the GEV distribution can be sensitive to record length for a mixed extreme climate, and can underestimate higher return levels.

The Gumbel scale parameter (and the slope of the Gumbel return level curve) has been used to indicate an increase in the frequency of extreme sea level events due to sea level rise (SLR), when assuming stationary EVD^34,35^. The steeper Gumbel slope of the TC events when compared to non-TC events, will result in SLR causing a greater increase of occurrence of non-TC extreme water level events than TC events. Hence, for locations such as the east coast of Queensland, Australia, where the estimated transition from non-TC to TC extreme storm tide water levels are for ARIs greater than 100 years^8^, it remains relevant to pay attention to the increase of non-TC extreme sea level events due to sea level rise^36,37^. However, this is not the case for locations such as Galveston, where the expected transition from non-TC to TC extreme water levels occurs at an ARI less than 5 years (Fig. 2a,b). For critical infrastructure which require a very low probability of return levels being exceeded, higher ARIs greater than 100 years that are driven by TCs must always be considered. MC methods applied to more tide gauge observations presents an opportunity to further validate and improve extreme event climatologies estimated by numerical modelling studies where observed TC events are abundant^7,8,27^.

The methodology of the MC EVD could be expanded to include the peaks-over-threshold approach, where all values over fixed thresholds are modelled (e.g.^5,23^). This should enable the use of data beyond just the annual maxima, improving inferential outcomes when such data is available. There are however challenges to overcome, such as the choice of thresholds in the mixed climate case, and the method for combining two tail models with potentially different thresholds. Any short-term correlation in the storm surge residual sea-level would also need to be accounted for, for example through the use of external clustering.

It would also be possible to expand the MC EVD using a hybrid approach^38^ with potentially different model forms for each aspect of the mixed climate. Here we use two Gumbel distributions with different location and scale parameters, which appears to work well for a large number of sites with potentially different behaviour. If the data record is large, then it may be possible to expand this using more flexible models with more parameters. However, there may be a lack of robustness in the maximum likelihood estimation if the information in the data is not sufficient, particularly if the model involves the estimation of distribution shape on both of the components.

Future work could include the effect of waves on extreme sea levels for open ocean sandy beaches^39–41^ and reef environments^42^ along with the future nonstationary changes to EVD from global climate model projections^24^. Future work could also replace the sea level residual with skew surge analysis which could be recombined with tide using convolution methods^43^ along with structural function approaches^41^ to define extreme water level populations. The mixed climate analysis for tide gauge extreme water levels can, for example, be applied to extreme wind-waves for coastal protection or for extreme winds for building design standards in tropical cyclone locations.

## Methods

### Data

Hourly tide gauge records were downloaded from the University of Hawaii Sea Level Centre (UHSLC)^13^. To obtain the storm tide height (sometimes referred to as the still water level as it does not include the effect of waves), the sea level rise, interannual and seasonal fluctuation were first removed from the records with a low pass filter, using the 30-day median. Hence storm-tide levels are relative to the 30-day median. To analyse the storm surge residual sea level, the predicted tide was computed for each year using 27 harmonic constituents in the R package “TideHarmonics”^44^ and removed from the record. A higher number of constituents was tested but showed little difference on the resulting residuals. There are a small number of missing values in the tide gauge records; these are not interpolated and remain missing in the storm surge residual sea level, with the subsequent annual maxima taken over the non-missing values.

Synthetic TC extreme water levels were sourced from previous studies^11,33^. The 1 in 50 and 1 in 2000 year average recurrence interval levels from the synthetic TC analysis^11^ were used with the Gumbel EVD (Eq. 3) to back solve the location and scale parameters.

The IBTRACs^45^ dataset was used to identify when a TC was located within 3 nautical degree radius of the tide gauge. Annual maximum values were then associated with a TC if a TC passed within a 24-h window. Fig. 1 maps the number of TC and non-TC tracks falling into two degree grid cells, noting track identification is significantly better post 1981^46,47^.

### Annual recurrence interval

All statistical analysis was conducted using R^48^. The method of annual maxima was used to evaluate extreme water levels in the tide gauge record. The GEV and Gumbel distributions were fitted with the ‘ismev’ R package^22^. To simulate $n$ years of annual maximum data from mixed extreme climate, the maximum value of a random sample from both the non-TC tide gauge record (${z}_{1}$) and synthetic TC record (${z}_{2}$) Gumbel EVD was repeated $n$ times using R’s “evd” package^49^. 90 percent confidence intervals around the maximum likelihood estimate (MLE) GEV or MC EVD were computed from the quantiles of 100 Monte Carlo simulations of the fitted model.

The mixed climate extreme value distribution (MC EVD) is formulated from two Gumbel distributions^50^. The Gumbel distribution estimate of water levels for a single population of extremes is,

$$z(\mathrm{A}RI)= \mu -\lambda \mathrm{log}\left(\frac{1}{ARI}\right),$$

where $z$ is the return water level corresponding to either the tide gauge (${z}_{1}$) or synthetic TC record (${z}_{2}$), $\mu$ is the Gumbel EVD location parameter, and $\lambda$ is the Gumbel EVD scale parameter, fitted to the hindcast maxima (with the annual mean value removed) using maximum likelihood. The annual recurrence interval ($\mathrm{A}RI$) is given by $-1/log(1-p)$, where $p$ is the annual exceedance probability. We define ARI as the average recurrence interval since it is approximately equal to 1/$p$ for small $p$. The intersection of two Gumbel EVDs, where ${z}_{1}={z}_{2}$ , for the non-TC (${z}_{1}$) and TC (${z}_{2}$) can be derived as,$\mathrm{exp}\left(-\frac{{\mu }_{2}-{\mu }_{1}}{{\lambda }_{2}-{\lambda }_{1}}\right)$.

### Maximum likelihood estimation

For any two independent random variables ${Z}_{1}$ and ${Z}_{2}$, we have ${F}_{Z}\left(z\right)=P\left\{\mathrm{max}({Z}_{1},{Z}_{2}) <z\right\}=P\left\{{Z}_{1}<z\right\}P\left\{{Z}_{2}<z\right\}={F}_{1}(z){F}_{2}(z)$ where ${F}_{j}\left(\cdot \right)$ is the distribution functions of ${Z}_{j} \mathrm{for} j=\mathrm{1,2}$. This means that the density function for the mixed climate, $\mathrm{max}({Z}_{1},{Z}_{2})$, is given by $\frac{d}{dz}{F}_{Z}\left(z\right)={f}_{1}\left(z\right){F}_{2}\left(z\right)+{f}_{2}\left(z\right){F}_{1}\left(z\right)$ where ${f}_{j}\left(\cdot \right)$ is the density functions of ${Z}_{j} \mathrm{for} j=\mathrm{1,2}$. The likelihood function for the maximum of two Gumbel (${\mu }_{j}{\lambda }_{j}$) distributions is therefore,

$$ \mathop \prod \limits_{i = 1}^{n} \left\{ {\frac{1}{{\lambda_{2} }}\exp \left( { - \exp \left( { - \left( {x_{i1} - \mu_{1} } \right)/\lambda_{1} } \right)} \right)\exp \left( {\left( {x_{i2} - \mu_{2} } \right)/\lambda_{2} - \exp \left( { - \left( {x_{i2} - \mu_{2} } \right)/\lambda_{2} } \right)} \right) + \frac{1}{{\lambda_{1} }}\exp \left( { - \exp \left( { - \left( {x_{i2} - \mu_{2} } \right)/\lambda_{2} } \right)} \right) \exp \left( {\left( {x_{i1} - \mu_{1} } \right)/\lambda_{1} - \exp \left( { - \left( {x_{i1} - \mu_{1} } \right)/\lambda_{1} } \right)} \right)} \right\} $$

and hence for data ${(x}_{i1},{x}_{i2}) for i = 1, \dots , n$, the maximum likelihood estimates of the parameters of the MC EVD (${\mu }_{1}{,\lambda }_{1},{\mu }_{2}{,\lambda }_{2}$) are obtained by maximizing the above. This procedure is available via the *fgumbelx* function in the R package “evd”^49^. In practice we maximize the logarithm of the likelihood over (${\mu }_{1},\mathrm{log}\left({\lambda }_{1}\right),\mathrm{log}\left({\mu }_{1}-{\mu }_{2}\right),\mathrm{log}\left({\lambda }_{2}\right))$, which implies the constraint ${\mu }_{1}\ge {\mu }_{2}$.The maximum of two Gumbel distributions with the same scale parameter is also a Gumbel distribution, and we can therefore make use of the first two probability weighted moments ${\beta }_{0}=E[Z]$ and ${{\beta }_{1}=E[ZF}_{Z}\left(z\right)]$ to derive the starting values

$${\lambda }_{1}^{0}={\lambda }_{2}^{0}=\frac{2{\beta }_{1}-{\beta }_{0}}{\mathrm{log}\left(2\right)},$$

$${\mu }_{1}^{0}={\beta }_{0}-{\lambda }_{1}^{0}\gamma -{\lambda }_{1}^{0}\mathrm{log}\left(2\right),$$

where $\gamma \approx 0.577$ is the Euler-Mascheroni constant, with ${\beta }_{0}$ and ${\beta }_{1}$ replaced by empirical estimates^51^. The starting value for $\mathrm{log}\left({\mu }_{2}-{\mu }_{1}\right)$ can be set as arbitrarily small.

For a given ARI, the estimate of the water level z(ARI) for a continuous MC EVD can be derived as the solution to,

$$\mathrm{exp}\left(-\frac{z-{\upmu }_{1}}{{\uplambda }_{1}}\right)+\mathrm{exp}\left(-\frac{z-{\upmu }_{2}}{{\uplambda }_{2}}\right)=1/\mathrm{ARI},$$

which can be found using a root-finding algorithm^52^ using the R package “evd”^49^.

## Supplementary Information

Supplementary Information.
