# Supplementary material for: Gauging mixed climate extreme value distributions in tropical cyclone regions
Source: Sci Rep. 2022 Mar 17;12:4626. doi: 10.1038/s41598-022-08382-y (PMC8931004; doi:10.1038/s41598-022-08382-y)
Supplement: Supplementary file 1 — Supplementary Information. [file 41598_2022_8382_MOESM1_ESM.docx]

Gauging Mixed Climate Extreme Value Distributions in Tropical Cyclone Regions

## Authors: O’Grady Stephenson McInnes.

Supplementary report.


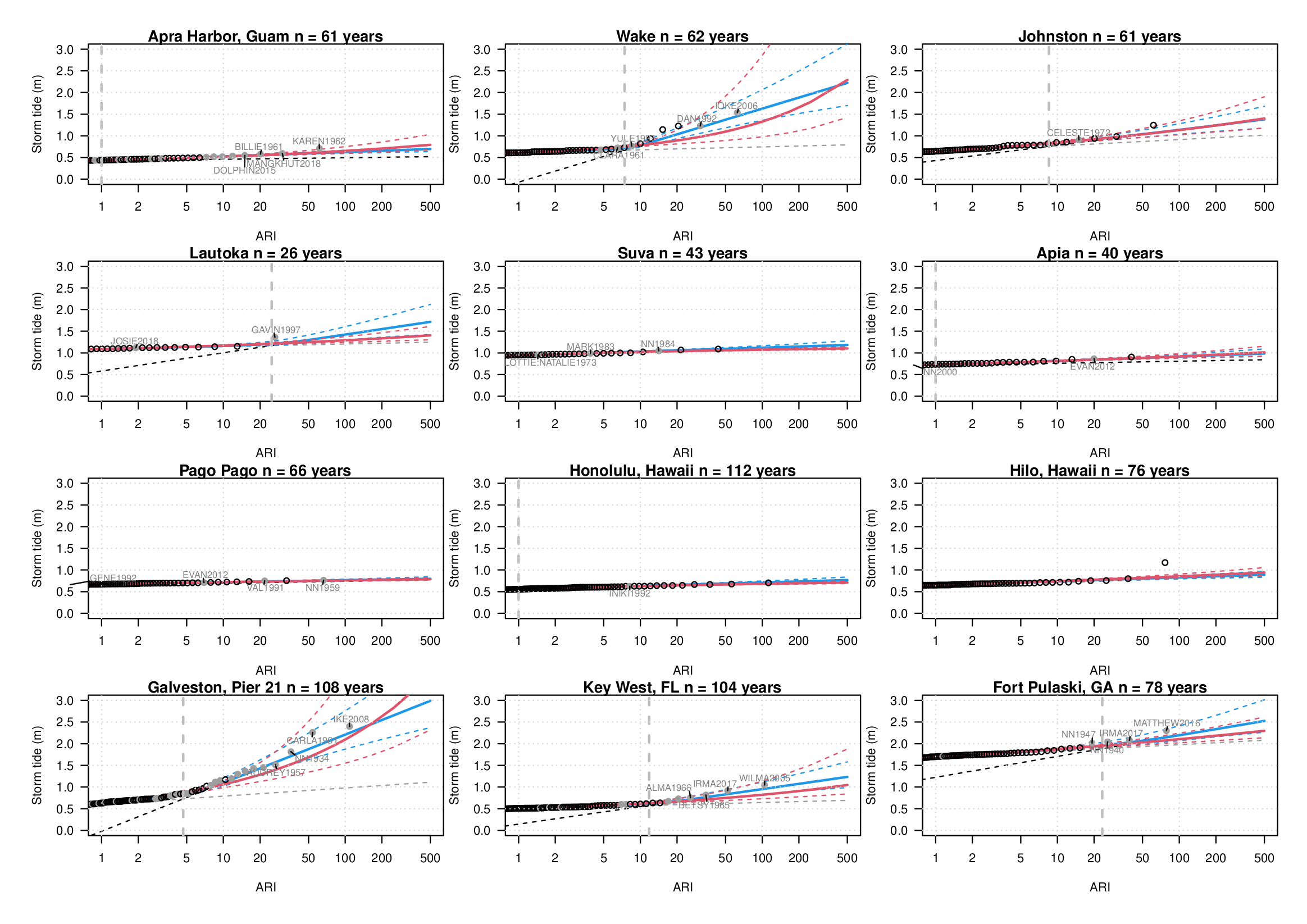


Figure S1 Tide gauge extreme storm tide empirically ranked annual maximum (black circles) ) and TC events (grey points) with fitted MC Gumbel (grey and black dashed lines), continuous MC (blue line) and GEV (red line) EVDs with 90% confidence intervals (dashed curves). Vertical thick grey dashed line indicates the intersection of the two MC Gumbel EVD. Top four IBTrACS storms are named EVD parameter values provided in Table S2.


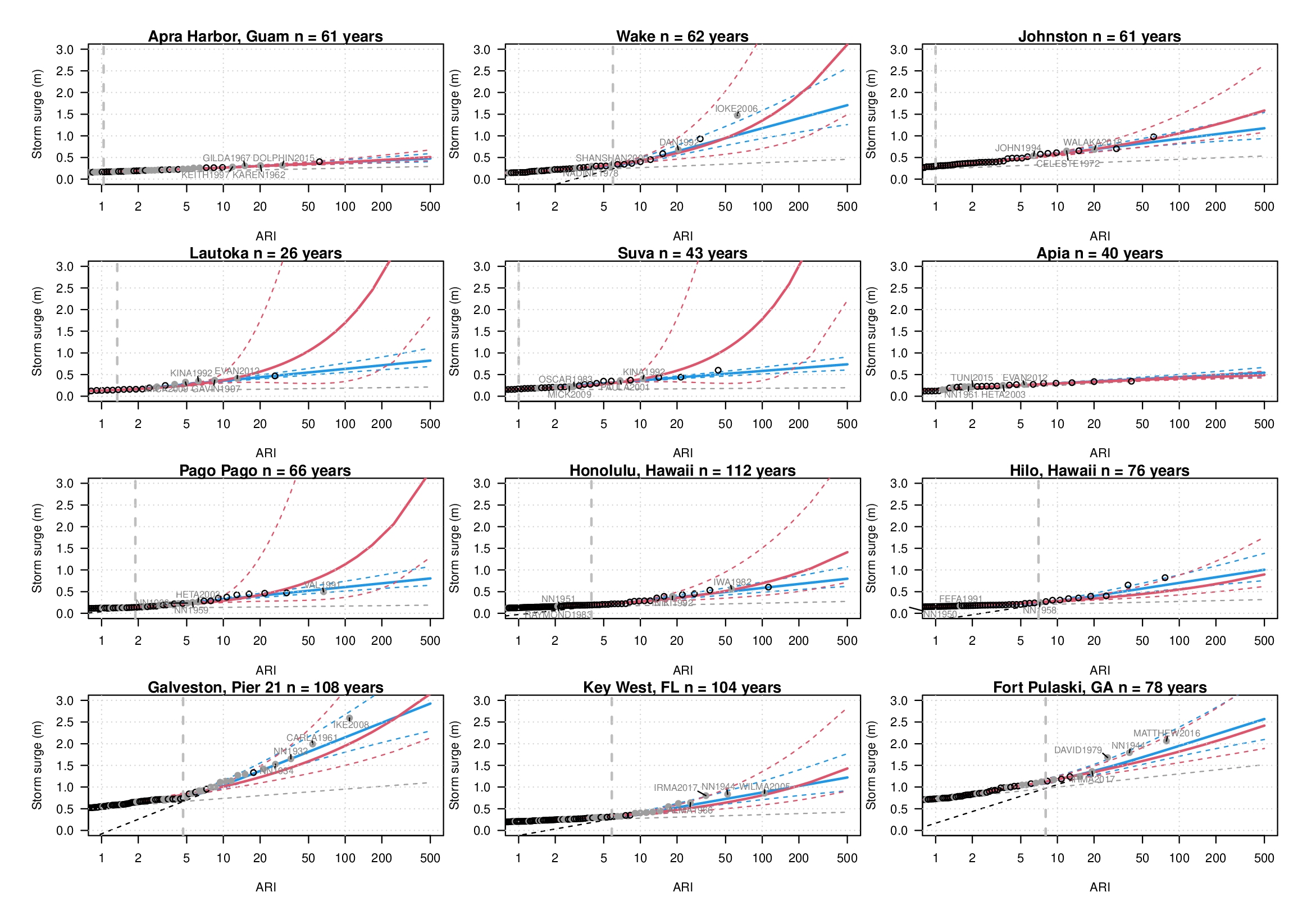


Figure S2 Tide gauge extreme storm surge empirically ranked annual maximum (black circles) ) and TC events (grey points) with fitted MC Gumbel (grey and black dashed lines), continuous MC (blue line) and GEV (red line) EVDs with 90% confidence intervals (dashed curves). Vertical thick grey dashed line indicates the intersection of the two MC Gumbel EVD. Top four IBTrACS storms are named EVD parameter values provided in Table S3.

Figure S3 Key West tide gauge storm surge (tide removed) EVD sensitivity to record length (n). Empirically ranked annual maximum storm tide (black circles) with fitted MC Gumbel (grey and black dashed lines), continuous MC (blue line) and GEV (red line) EVDs using the first n years of annual maxima , a) n = 25,b) n = 50, c) n= 75 and d) n = 108. Vertical thick grey dashed line indicates the intersection of the two MC Gumbel EVDs.

Figure S4 Random sampling of the mixed-climate EVDs for record length (n) equals 40 years (top left), 100 years (top right) and 10000 years (bottom left) for Suva.


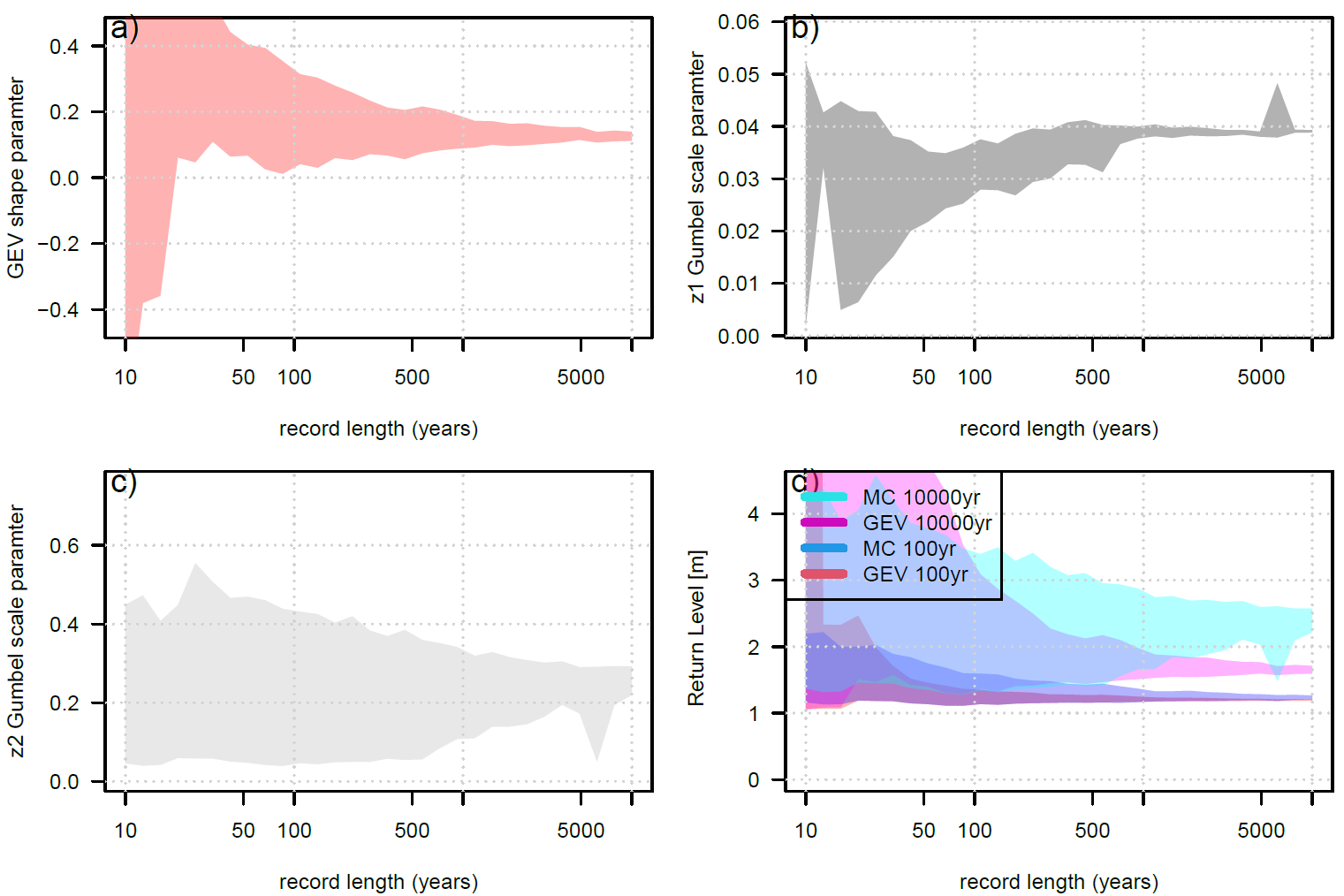


Figure S5 stability plot for the generalised (GEV) and mixed climate (MC) EVD parameters and return levels for increasing record length (random samples) for storm tide at Suva, Fiji. X-axis on a log scale. 90% confidence bounds calculated from 100 Monte Carlo simulations for each record length.

Figure S6 Random sampling of the mixed-climate EVDs for record length (n) equals 40 years (top left), 100 years (top right) and 10000 years (bottom left) for Apia.


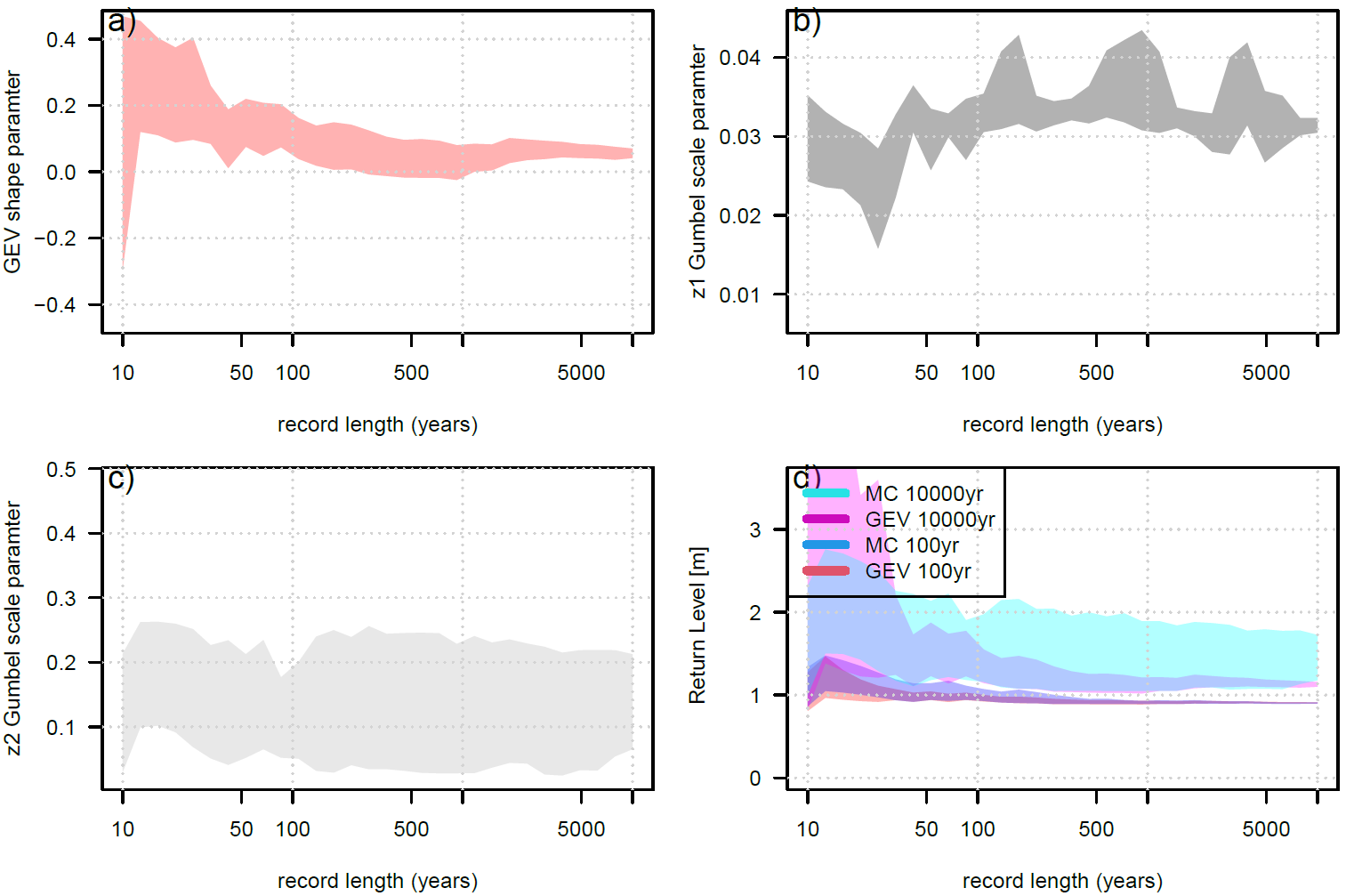


Figure S7 stability plot for the generalised (GEV) and mixed climate (MC) EVD parameters and return levels for increasing record length (random samples) for storm tide at Apia, Samoa. X-axis on a log scale. 90% confidence bounds calculated from 100 Monte Carlo simulations for each record length.

Table S1 Mixed climate location (mu) and scale (lam) parameters for the tide gauge and synthetically simulated storm tide.

|  | z1_mu | z1_lam | z2_mu | z2_lam | ARII | z1_n |
| --- | --- | --- | --- | --- | --- | --- |
| Lautoka | 1.092029 | 0.031764 | -0.64 | 0.604 | 20.62955 | 26 |
| Suva | 0.943863 | 0.038532 | -0.06 | 0.274 | 71.08363 | 43 |
| Apia | 0.734919 | 0.032771 | -0.052 | 0.18 | 209.6417 | 40 |

Table S2 storm tide mixed climate and GEV parameters for study locations (Figure 1). See Figure S1 for EVD plots.

| Station country | Station Name | n years | Longitude | Latitude | $\mu_{1}$ | $\lambda_{1}$ | $\mu_{2}$ | $\lambda_{2}$ | GEV location | GEV scale | GEV shape |
| --- | --- | --- | --- | --- | --- | --- | --- | --- | --- | --- | --- |
| United States of America (the) | Apra Harbor, Guam | 61 | 144.65 | 13.433 | 0.4231 | 0.0439 | 0.4231 | 0.0155 | 0.4403 | 0.0268 | 0.2176 |
| United States of America (the) | Wake | 62 | 166.617 | 19.283 | 0.6109 | 0.0291 | -0.0697 | 0.3686 | 0.614 | 0.0407 | 0.4918 |
| United States of America (the) | Johnston | 61 | 190.47 | 16.738 | 0.6214 | 0.0635 | 0.4302 | 0.1527 | 0.6385 | 0.0753 | 0.146 |
| Fiji | Lautoka | 26 | 177.438 | -17.605 | 1.0878 | 0.0256 | 0.5835 | 0.1824 | 1.0891 | 0.0293 | 0.1636 |
| Fiji | Suva | 43 | 178.427 | 178.428 | 0.9172 | 0.0385 | 0.9172 | 0.0385 | 0.1149 | 507205.8 | 0.9468 |
| Samoa | Apia | 40 | 188.25 | 188.243 | 0.712 | 0.044 | 0.712 | 0.021 | 0.1311 | 19433.1 | 0.733 |
| American Samoa | Pago Pago | 66 | 189.317 | -14.283 | 0.6661 | 0.0201 | 0.6472 | 0.0201 | 0.6731 | 0.0202 | -0.0361 |
| United States of America (the) | Honolulu, Hawaii | 112 | 202.133 | 21.307 | 0.5301 | 0.0343 | 0.5301 | 0.0343 | 0.5561 | 0.0352 | -0.1217 |
| United States of America (the) | Hilo, Hawaii | 76 | 204.933 | 19.733 | 0.623 | 0.0387 | 0.623 | 0.0387 | 0.6482 | 0.0379 | 0.0683 |
| United States of America (the) | Galveston, Pier 21 | 108 | 265.207 | 29.31 | 0.6008 | 0.0822 | -0.021 | 0.4847 | 0.625 | 0.1197 | 0.3739 |
| United States of America (the) | Key West, FL | 104 | 278.192 | 24.553 | 0.5009 | 0.0309 | 0.1441 | 0.1755 | 0.5042 | 0.0376 | 0.2411 |
| United States of America (the) | Fort Pulaski, GA | 78 | 279.098 | 32.033 | 1.6826 | 0.0641 | 1.2246 | 0.2095 | 1.69 | 0.0724 | 0.0927 |

Table S3 storm surge mixed climate and GEV parameters for study locations (Figure 1). See Figure S2 for EVD plots.

| Station country | Station Name | n years | Longitude | Latitude | $\mu_{1}$ | $\lambda_{1}$ | $\mu_{2}$ | $\lambda_{2}$ | GEV location | GEV scale | GEV shape |
| --- | --- | --- | --- | --- | --- | --- | --- | --- | --- | --- | --- |
| United States of America (the) | Apra Harbor, Guam | 61 | 144.65 | 13.433 | 0.1431 | 0.0241 | 0.1419 | 0.054 | 0.1676 | 0.0372 | 0.1195 |
| United States of America (the) | Wake | 62 | 166.617 | 19.283 | 0.1533 | 0.049 | -0.35 | 0.3311 | 0.1582 | 0.0604 | 0.5297 |
| United States of America (the) | Johnston | 61 | 190.47 | 16.738 | 0.2364 | 0.048 | 0.2364 | 0.1511 | 0.2961 | 0.0912 | 0.2359 |
| Fiji | Lautoka | 26 | 177.438 | -17.605 | 0.1147 | 0.0157 | 0.084 | 0.1188 | 0.1322 | 0.0389 | 0.7455 |
| Fiji | Suva | 43 | 178.427 | 178.428 | 0.1461 | 0.0082 | 0.1459 | 0.0951 | 0.0893 | 0.8461 | 0.1667 |
| Samoa | Apia | 40 | 188.25 | 188.243 | 0.1103 | 0.0627 | 0.1103 | 0.0627 | 0.1298 | 1682954 | 0.1561 |
| American Samoa | Pago Pago | 66 | 189.317 | -14.283 | 0.1081 | 0.0128 | 0.0375 | 0.1234 | 0.1194 | 0.0303 | 0.6898 |
| United States of America (the) | Honolulu, Hawaii | 112 | 202.133 | 21.307 | 0.122 | 0.0243 | -0.0274 | 0.1329 | 0.129 | 0.0343 | 0.4703 |
| United States of America (the) | Hilo, Hawaii | 76 | 204.933 | 19.733 | 0.1485 | 0.027 | -0.1655 | 0.1884 | 0.1537 | 0.037 | 0.3244 |
| United States of America (the) | Galveston, Pier 21 | 108 | 265.207 | 29.31 | 0.5218 | 0.0935 | -0.0795 | 0.4838 | 0.5526 | 0.136 | 0.3124 |
| United States of America (the) | Key West, FL | 104 | 278.192 | 24.553 | 0.1994 | 0.0354 | -0.1175 | 0.2153 | 0.2066 | 0.048 | 0.3816 |
| United States of America (the) | Fort Pulaski, GA | 78 | 279.098 | 32.033 | 0.6962 | 0.1319 | 0.166 | 0.3869 | 0.7306 | 0.1629 | 0.1525 |

Table S4 IBTrACS technical documentation of storm track identification, and our broad interpretation of either Thunderstorm or TC. <https://www.ncdc.noaa.gov/ibtracs/pdf/IBTrACS_version4_Technical_Details.pdf>

| USA_STATUS | USA_Discription | Our Interpretation |
| --- | --- | --- |
| DB | disturbance | non-TC |
| TD | tropical depression | non-TC |
| TS | tropical storm | non-TC |
| TY | typhoon | Tropical Cyclone |
| ST | super typhoon | Tropical Cyclone |
| TC | tropical cyclone | Tropical Cyclone |
| HU,HR | hurricane | Tropical Cyclone |
| SD | subtropical depression | non-TC |
| SS | subtropical storm | non-TC |
| EX | extratropical systems | non-TC |
| PT | post tropical | non-TC |
| IN | inland | non-TC |
| DS | dissipating | non-TC |
| LO | low | non-TC |
| WV | tropical wave | non-TC |
| ET | extrapolated | non-TC |
| MD | monsoon depression | non-TC |
| XX | unknown. | non-TC |

Table S5 Galveston storm tide annual max with IBTrACS identified storms. The US_STATUS storm classifications abbreviations are detailed in Table S4 and are the unique classifications that have been made within 24 hour window of the water level peak, hence more than one classification. “NN” indicates unnamed track.

|  | Storm tide [m] | Date | IBTRACS_SID | name | USA_STATUS |
| --- | --- | --- | --- | --- | --- |
| 1 | 2.4 | 2008091302 | 2008245N17323 | IKE2008 | HU |
| 2 | 2.26 | 1961091123 | 1961247N13283 | CARLA1961 | HU |
| 3 | 1.82 | 1934072511 | 1934203N32281 | NN1934 | HU |
| 4 | 1.51 | 1957062711 | 1957176N21267 | AUDREY1957 | HU |
| 5 | 1.45 | 1942083005 | 1942236N15286 | NN1942 | HU |
| 6 | 1.43 | 1983081808 | 1983228N27270 | ALICIA1983 | HU TS |
| 7 | 1.37 | 1932081403 | 1932225N22270 | NN1932 | HU |
| 8 | 1.3 | 2003071512 | 2003188N11307 | CLAUDETTE2003 | TS HU |
| 9 | 1.2 | 1941092403 | 1941261N24272 | NN1941 | HU TS |
| 10 | 1.17 | 1919091513 | NA | NA |  |
| 11 | 1.15 | 1963091710 | 1963260N27266 | CINDY1963 | TS |
| 12 | 1.12 | 1949100406 | 1949270N13270 | NN1949 | HU TS |
| 13 | 1.04 | 1998091014 | 1998252N26266 | FRANCES1998 | TS |
| 14 | 1.02 | 1969021414 | NA | NA |  |
| 15 | 0.96 | 1980080917 | NA | NA |  |
| 16 | 0.93 | 1906101414 | NA | NA |  |
| 17 | 0.93 | 1933070609 | NA | NA |  |
| 18 | 0.89 | 2006101609 | NA | NA |  |
| 19 | 0.85 | 1971112308 | NA | NA |  |
| 20 | 0.85 | 1973090420 | 1973245N21274 | DELIA1973 | TS |
| 21 | 0.85 | 1989101602 | 1989286N19268 | JERRY1989 | TS HU TD |
| 22 | 0.84 | 1947052320 | NA | NA |  |
| 23 | 0.84 | 2001060521 | 2001157N28265 | ALLISON2001 | TS TD |
| 24 | 0.83 | 1923101609 | NA | NA |  |
| 25 | 0.81 | 2002090711 | 2002249N28266 | FAY2002 | TS TD |
| 26 | 0.8 | 2015061610 | 2015167N27266 | BILL2015 | TS |
| 27 | 0.78 | 1920112623 | NA | NA |  |
| 28 | 0.77 | 1944120507 | NA | NA |  |
| 29 | 0.77 | 1997040508 | NA | NA |  |
| 30 | 0.75 | 1948010102 | NA | NA |  |
| 31 | 0.74 | 1958090607 | 1958242N13302 | ELLA1958 | TS TD |
| 32 | 0.73 | 1912061107 | NA | NA |  |
| 33 | 0.73 | 1938101712 | 1938284N16273 | NN1938 | TS |
| 34 | 0.73 | 1977090114 | NA | NA |  |
| 35 | 0.73 | 2017062117 | 2017171N24271 | CINDY2017 | TS |
| 36 | 0.72 | 1929041314 | NA | NA |  |
| 37 | 0.72 | 1946110407 | NA | NA |  |
| 38 | 0.72 | 1970030707 | NA | NA |  |
| 39 | 0.72 | 1991011506 | NA | NA |  |
| 40 | 0.72 | 1996100607 | NA | NA |  |
| 41 | 0.71 | 1952071713 | NA | NA |  |
| 42 | 0.71 | 1986122305 | NA | NA |  |
| 43 | 0.71 | 1995073100 | 1995210N26273 | DEAN1995 | TD TS |
| 44 | 0.7 | 1936052416 | NA | NA |  |
| 45 | 0.7 | 1967092100 | NA | NA |  |
| 46 | 0.7 | 1993062011 | NA | NA |  |
| 47 | 0.69 | 1910091411 | 1910248N17302 | NN1910 | HU |
| 48 | 0.69 | 1927041419 | NA | NA |  |
| 49 | 0.69 | 1940010623 | NA | NA |  |
| 50 | 0.69 | 1953042916 | NA | NA |  |
| 51 | 0.69 | 1954111406 | NA | NA |  |
| 52 | 0.68 | 1988043008 | NA | NA |  |
| 53 | 0.68 | 2007112423 | NA | NA |  |
| 54 | 0.67 | 2000012800 | NA | NA |  |
| 55 | 0.67 | 2004111609 | NA | NA |  |
| 56 | 0.67 | 2005092313 | 2005261N21290 | RITA2005 | HU |
| 57 | 0.67 | 2013010920 | NA | NA |  |
| 58 | 0.67 | 2016120309 | NA | NA |  |
| 59 | 0.66 | 1918122001 | NA | NA |  |
| 60 | 0.66 | 1924032000 | NA | NA |  |
| 61 | 0.66 | 1935121200 | NA | NA |  |
| 62 | 0.66 | 1960010100 | NA | NA |  |
| 63 | 0.66 | 1982051323 | NA | NA |  |
| 64 | 0.66 | 2010011600 | NA | NA |  |
| 65 | 0.65 | 1921062213 | 1921167N17278 | NN1921 | HU TS |
| 66 | 0.65 | 1978011201 | NA | NA |  |
| 67 | 0.64 | 1964100308 | NA | NA |  |
| 68 | 0.63 | 1913042313 | NA | NA |  |
| 69 | 0.63 | 1955020500 | NA | NA |  |
| 70 | 0.63 | 1962042717 | NA | NA |  |
| 71 | 0.62 | 1992110114 | NA | NA |  |
| 72 | 0.62 | 2011090308 | 2011245N27269 | LEE2011 | TS SS |
| 73 | 0.61 | 1908091717 | 1908261N25270 | NN1908 | TS |
| 74 | 0.61 | 1931032617 | NA | NA |  |
| 75 | 0.61 | 1968110607 | NA | NA |  |
| 76 | 0.61 | 1990110903 | NA | NA |  |
| 77 | 0.61 | 2018110104 | NA | NA |  |
| 78 | 0.6 | 1930113005 | NA | NA |  |
| 79 | 0.6 | 1945040114 | NA | NA |  |
| 80 | 0.6 | 1974121102 | NA | NA |  |
| 81 | 0.6 | 1985081510 | 1985224N18279 | DANNY1985 | HU |
| 82 | 0.59 | 1981060514 | 1981154N19266 | NN1981 | TD |
| 83 | 0.58 | 1911021101 | NA | NA |  |
| 84 | 0.58 | 1951032815 | NA | NA |  |
| 85 | 0.58 | 1975122500 | NA | NA |  |
| 86 | 0.57 | 1904012201 | NA | NA |  |
| 87 | 0.57 | 1914102510 | NA | NA |  |
| 88 | 0.57 | 1922060917 | NA | NA |  |
| 89 | 0.57 | 1937101720 | NA | NA |  |
| 90 | 0.57 | 1939071217 | NA | NA |  |
| 91 | 0.57 | 1950100306 | NA | NA |  |
| 92 | 0.57 | 1956011813 | NA | NA |  |
| 93 | 0.57 | 1959072501 | 1959204N28269 | DEBRA1959 | HU TS |
| 94 | 0.57 | 2014112307 | NA | NA |  |
| 95 | 0.56 | 1907122202 | NA | NA |  |
| 96 | 0.55 | 1917050611 | NA | NA |  |
| 97 | 0.55 | 1966050612 | NA | NA |  |
| 98 | 0.54 | 1909072215 | 1909195N12300 | NN1909 | TD |
| 99 | 0.54 | 1915120800 | NA | NA |  |
| 100 | 0.54 | 1928110101 | NA | NA |  |
| 101 | 0.53 | 1905042417 | NA | NA |  |
| 102 | 0.52 | 1999122106 | NA | NA |  |
| 103 | 0.52 | 2012032009 | NA | NA |  |
| 104 | 0.51 | 1926050606 | NA | NA |  |
| 105 | 0.51 | 1987111616 | NA | NA |  |
| 106 | 0.49 | 1965092109 | NA | NA |  |
| 107 | 0.48 | 1994122821 | NA | NA |  |
| 108 | 0.47 | 1925060922 | NA | NA |  |

Table S6 Gavleston storm surge annual max with IBTrACS identified storms. The US_STATUS storm classifications abbreviations are detailed in Table S4 and are the unique classifications that have been made within 24 hour window of the water level peak, hence more than one classification. “NN” indicates unnamed track.

|  | Storm surge[m] | Date | IBTRACS_SID | name | USA_STATUS |
| --- | --- | --- | --- | --- | --- |
| 1 | 2.59 | 2008091302 | 2008245N17323 | IKE2008 | HU |
| 2 | 1.99 | 1961091104 | 1961247N13283 | CARLA1961 | HU |
| 3 | 1.65 | 1932081403 | 1932225N22270 | NN1932 | HU |
| 4 | 1.53 | 1934072511 | 1934203N32281 | NN1934 | HU |
| 5 | 1.42 | 1942083005 | 1942236N15286 | NN1942 | HU |
| 6 | 1.33 | 1969021414 | NA | NA |  |
| 7 | 1.3 | 1983081807 | 1983228N27270 | ALICIA1983 | HU TS |
| 8 | 1.27 | 1957062711 | 1957176N21267 | AUDREY1957 | HU |
| 9 | 1.14 | 1941092404 | 1941261N24272 | NN1941 | HU TS |
| 10 | 1.14 | 2003071507 | 2003188N11307 | CLAUDETTE2003 | TS HU |
| 11 | 1.11 | 1949100406 | 1949270N13270 | NN1949 | HU TS |
| 12 | 1 | 1963091711 | 1963260N27266 | CINDY1963 | TS |
| 13 | 1 | 1973090420 | 1973245N21274 | DELIA1973 | TS |
| 14 | 0.95 | 1919091513 | NA | NA |  |
| 15 | 0.92 | 2005092321 | 2005261N21290 | RITA2005 | HU |
| 16 | 0.91 | 1998091014 | 1998252N26266 | FRANCES1998 | TS |
| 17 | 0.88 | 1906101414 | NA | NA |  |
| 18 | 0.85 | 1947082422 | 1947231N23278 | NN1947 | HU TS |
| 19 | 0.84 | 1980080917 | NA | NA |  |
| 20 | 0.77 | 2001060522 | 2001157N28265 | ALLISON2001 | TS TD |
| 21 | 0.75 | 1944120516 | NA | NA |  |
| 22 | 0.73 | 1958090522 | 1958242N13302 | ELLA1958 | TS |
| 23 | 0.73 | 1997042602 | NA | NA |  |
| 24 | 0.72 | 1933080401 | NA | NA |  |
| 25 | 0.72 | 1938050712 | NA | NA |  |
| 26 | 0.72 | 1989101602 | 1989286N19268 | JERRY1989 | TS HU TD |
| 27 | 0.72 | 2002090703 | 2002249N28266 | FAY2002 | TS TD |
| 28 | 0.72 | 2006101609 | NA | NA |  |
| 29 | 0.72 | 2015061603 | 2015167N27266 | BILL2015 | TS |
| 30 | 0.71 | 1909050705 | NA | NA |  |
| 31 | 0.71 | 1918011318 | NA | NA |  |
| 32 | 0.71 | 1967092017 | NA | NA |  |
| 33 | 0.71 | 1970020115 | NA | NA |  |
| 34 | 0.7 | 1923101522 | NA | NA |  |
| 35 | 0.7 | 1986062608 | 1986175N26273 | BONNIE1986 | HU TS |
| 36 | 0.69 | 1991011817 | NA | NA |  |
| 37 | 0.68 | 1907111713 | NA | NA |  |
| 38 | 0.68 | 1962112715 | NA | NA |  |
| 39 | 0.68 | 1977090117 | NA | NA |  |
| 40 | 0.67 | 1971112308 | NA | NA |  |
| 41 | 0.67 | 1993062001 | NA | NA |  |
| 42 | 0.66 | 1927041419 | NA | NA |  |
| 43 | 0.66 | 1995073100 | 1995210N26273 | DEAN1995 | TD TS |
| 44 | 0.66 | 2017082904 | 2017228N14314 | HARVEY2017 | TS |
| 45 | 0.65 | 1992011208 | NA | NA |  |
| 46 | 0.65 | 2000012800 | NA | NA |  |
| 47 | 0.64 | 2016030920 | NA | NA |  |
| 48 | 0.63 | 1988043005 | NA | NA |  |
| 49 | 0.62 | 1948010102 | NA | NA |  |
| 50 | 0.62 | 1996111620 | NA | NA |  |
| 51 | 0.61 | 1955011811 | NA | NA |  |
| 52 | 0.6 | 1908091718 | 1908261N25270 | NN1908 | TS |
| 53 | 0.6 | 1912061101 | NA | NA |  |
| 54 | 0.6 | 1940021710 | NA | NA |  |
| 55 | 0.6 | 2010020414 | NA | NA |  |
| 56 | 0.59 | 1915120715 | NA | NA |  |
| 57 | 0.59 | 2007112419 | NA | NA |  |
| 58 | 0.59 | 2013040305 | NA | NA |  |
| 59 | 0.58 | 1952071719 | NA | NA |  |
| 60 | 0.58 | 1975122500 | NA | NA |  |
| 61 | 0.58 | 1982051323 | NA | NA |  |
| 62 | 0.57 | 1924032000 | NA | NA |  |
| 63 | 0.57 | 2011090300 | 2011245N27269 | LEE2011 | TS |
| 64 | 0.57 | 2012032016 | NA | NA |  |
| 65 | 0.56 | 1920120612 | NA | NA |  |
| 66 | 0.55 | 1930113005 | NA | NA |  |
| 67 | 0.55 | 1959072510 | 1959204N28269 | DEBRA1959 | HU TS |
| 68 | 0.55 | 1968120102 | NA | NA |  |
| 69 | 0.55 | 1990110902 | NA | NA |  |
| 70 | 0.55 | 2018102503 | NA | NA |  |
| 71 | 0.54 | 1945040118 | NA | NA |  |
| 72 | 0.53 | 1946110407 | NA | NA |  |
| 73 | 0.53 | 1964011612 | NA | NA |  |
| 74 | 0.53 | 1965022407 | NA | NA |  |
| 75 | 0.53 | 1985102705 | NA | NA |  |
| 76 | 0.52 | 1929041406 | NA | NA |  |
| 77 | 0.52 | 1936052510 | NA | NA |  |
| 78 | 0.52 | 1953042916 | NA | NA |  |
| 79 | 0.52 | 1960121020 | NA | NA |  |
| 80 | 0.52 | 1978020804 | NA | NA |  |
| 81 | 0.52 | 1987111616 | NA | NA |  |
| 82 | 0.51 | 1910091412 | 1910248N17302 | NN1910 | HU |
| 83 | 0.51 | 1954111417 | NA | NA |  |
| 84 | 0.51 | 1974022114 | NA | NA |  |
| 85 | 0.5 | 1956011813 | NA | NA |  |
| 86 | 0.5 | 2004110212 | NA | NA |  |
| 87 | 0.49 | 1911021111 | NA | NA |  |
| 88 | 0.49 | 1913042405 | NA | NA |  |
| 89 | 0.49 | 1922012916 | NA | NA |  |
| 90 | 0.49 | 1939071201 | NA | NA |  |
| 91 | 0.48 | 1950032707 | NA | NA |  |
| 92 | 0.47 | 1935123120 | NA | NA |  |
| 93 | 0.47 | 1937121617 | NA | NA |  |
| 94 | 0.47 | 1999031311 | NA | NA |  |
| 95 | 0.46 | 1921041306 | NA | NA |  |
| 96 | 0.46 | 1928040602 | NA | NA |  |
| 97 | 0.46 | 1931032616 | NA | NA |  |
| 98 | 0.46 | 1981060509 | 1981154N19266 | NN1981 | TD |
| 99 | 0.45 | 1905021113 | NA | NA |  |
| 100 | 0.45 | 1925011523 | NA | NA |  |
| 101 | 0.45 | 1926050606 | NA | NA |  |
| 102 | 0.45 | 2014101319 | NA | NA |  |
| 103 | 0.44 | 1904012201 | NA | NA |  |
| 104 | 0.44 | 1951032801 | NA | NA |  |
| 105 | 0.43 | 1966050607 | NA | NA |  |
| 106 | 0.42 | 1994011701 | NA | NA |  |
| 107 | 0.41 | 1914102511 | NA | NA |  |
| 108 | 0.34 | 1917050701 | NA | NA |  |
